# Efficacy and safety evaluation of cross-reactive Fibroblast activation protein scFv-based CAR-T cells

**DOI:** 10.3389/fimmu.2024.1433679

**Published:** 2024-07-17

**Authors:** Wenhao Niu, Binchen Wang, Yirui Zhang, Chaomin Wang, Jing Cao, Jiali Li, Yong He, Ping Lei

**Affiliations:** ^1^ Department of Immunology, School of Basic Medicine, Tongji Medical College, Huazhong University of Science and Technology, Wuhan, China; ^2^ Department of Nuclear Medicine, Zhongnan Hospital of Wuhan University, Wuhan, China; ^3^ Department of Clinical Laboratory, National Clinical Research Center for Cancer, Key laboratory of Cancer Prevention and Therapy, Tianjin’s Clinical Research Center for Cancer, Tianjin Medical University Cancer Institute and Hospital, Tianjin, China

**Keywords:** fibroblast activation protein, cancer-associated fibroblasts, chimeric antigen receptor, on-target off-tumor toxicity, solid tumor

## Abstract

**Introduction:**

Fibroblast activation protein (FAP) overexpression on cancer-associated fibroblasts (CAFs) is associated with poor prognosis and worse clinical outcomes. Selective ablation of pro-tumorgenic FAP^+^ stromal cells with CAR-T cells may be a new therapeutic strategy. However, the clinical use of FAP-CAR T cells is suggested to proceed with caution for occasional poor efficacy and induction of on-target off-tumor toxicity (OTOT), including lethal osteotoxicity and cachexia. Hence, more investigations and preclinical trials are required to optimize the FAP-CAR T cells and to approve their safety and efficacy.

**Methods:**

In this study, we designed second-generation CAR T cells targeting FAP with 4-1BB as a co-stimulatory molecule, and tested their cytotoxicity against FAP-positive cells (hFAP-HT1080 cells and a variety of primary CAFs) *in vitro* and in Cell line-derived xenograft (CDX) and a patient-derived xenograft (PDX) model.

**Results:**

Results showed that our FAP-CAR T cells were powerfully potent in killing human and murine FAP-positive tumor cells and CAFs in multiple types of tumors in BALB/c and C57BL/6 mice and in patient-derived xenografts (PDX) model. And they were proved to be biologically safe and exhibit low-level OTOT.

**Discussion:**

Taken together, the human/murine cross-reactive FAP-CAR T cells were powerfully potent in killing human and murine FAP positive tumor cells and CAFs. They were biologically safe and exhibit low-level OTOT, warranting further clinical investigation into our FAP-CAR T cells.

## Introduction

Advanced solid tumors are largely composed of desmoplastic stroma. Cancer-associated fibroblasts (CAFs) are fundamental component in it and can contribute to up to 90% of the solid tumor mass (1). CAFs associate with biomechanical changes in tumor stroma and take important parts in shaping the extracellular matrix into a fibrotic ECM, which would form a barrier to drug delivery and immune surveillance (2). In addition, CAFs establish immune crosstalk by producing chemokines and cytokines, exosomes and metabolites, recruiting immune cells with inhibitory functions in the tumor stroma, thereby interfering with T cell function and promoting immune escape (3, 4). Because of these pro-oncogenic functions, deletion of CAFs have long been considered an attractive tumor therapeutic approach, especially in fibroblast-rich tumors (5).

CAFs are heterogeneous cells. Different subtypes of CAFs have been proposed to have disparate effects on tumor establishment, growth and progression in the setting of solid tumors (5). Therefore, when choosing the targeted protein, it is important to consider which fibroblast cell subpopulation is going to be depleted. Given this, fibroblast activation protein (FAP) has been proposed as a potentially good target. FAP, as a surface serine protease, is heavily expressed in a subset of pro-tumoral fibroblasts in more than 90% of common epithelial cancers, including colorectal, breast, pancreatic, skin, and lung tumors, whereas expression by normal organs is highly restricted (6–8). FAP has been reported to influence tumor growth via promoting tumor proliferation, invasion, angiogenesis, epithelial-to-mesenchymal transition, stem cell promotion, immunosuppression and drug resistance. Its overexpression on CAFs is associated with poor prognosis and worse clinical outcomes (9).

There have been many efforts to exploit FAP biology clinically, including peptides, antibodies, nanoparticles and small molecule inhibitors targeting FAP (6, 10, 11). Selective ablation of pro-tumorgenic FAP^+^ stromal cells with CAR-T cells may be a new therapeutic strategy (7, 12). However, the clinical use of FAP-CAR T cells is suggested to proceed with caution. One reason is that FAP-CAR T cells were reported to fail to regulate tumor growth, and induce on-target off-tumor toxicity (OTOT), including lethal osteotoxicity and cachexia (13). Gulati et al. suggested that the Lck binding moiety in CD28 costimulatory moiety could influence the efficacy of FAP-CAR T cells (14). And large-cohort clinical trials revealed that the rates of neurological toxicities have been higher with CD28-costimulated CARs than with 4-1BB-costimulated ones (15), although this finding was probably the result of a combination of factors rather than due to CD28 signaling alone. Hence, these challenges require further investigations regarding the optimization of the FAP-CAR T cells, and more preclinical trials to approve their safety and efficacy for reasonable prediction of the expected human response.

In this study, we generated CAR T cells with cytoplasmic domain combinations of 4-1BB instead of CD28 that can target both human FAP (hFAP) and mouse FAP (mFAP), and detected the killing function of FAP-CAR T on FAP^+^ tumor cells as well as human and murine primary CAFs *in vitro* and *in vivo*. In addition, we predicted the OTOT effect of CAR T treatment in a mouse model, which can reflect the possibility of OTOT occurrence in clinical therapy with CAR T to a certain extent. Results showed that the FAP-CAR T cells were powerfully potent in killing human and murine FAP-positive tumor cells and CAFs, and were biologically safe and exhibited low-level OTOT, warranting further clinical investigation into our FAP-CAR T cells.

## Materials and methods

### Cell culture

HT1080, U87MG, 4T1 and Panc02 cell lines were preserved in our lab. hFAP-HT1080 cell line was constructed and preserved in our laboratory (16). mFAP-HT1080, mFAP-Panc02 cell lines were established accordingly by amplifying murine *FAP* from mouse skin tissue. Human embryonic kidney 293T cells were obtained from the Cell Bank of Chinese Academy of Sciences (Shanghai, China). Immortalized human pancreatic tumor fibroblasts (PFs) were purchased from Wuhan Saios Biotechnology Co. Cells were cultured in DMEM or MEM (Gibco, USA) supplemented with 10% FBS (Shanghai Sangon Biotech, China).

Human heparinized peripheral blood samples were obtained from healthy donors with approval from the Medical Ethics Committee of Tongji Medical College. PBMC were isolated by Ficoll density centrifugation and cultured in X-VIVO 15 Medium (Lonza, Walkersville, USA) supplemented with 5% FBS.

Murine splenic T cells were enriched from BALB/c or C57BL/6 mice using untouched/negative isolation kits (Stem Cell). Cells were cultured in RPM1 1640 medium supplemented with 10% FBS, 1 mM sodium pyruvate, 1 mM HEPES and 50 μM β-mercaptoethanol.

### Isolation of primary CAFs

Human primary CAFs were freshly isolated from surgically resected tumor tissues as previous reports (17–19). Murine primary CAFs were isolated from KPC mouse (presented by Prof. Jun Zhao, Huazhong University of Science and Technology, Wuhan, China). In brief, the tumors were minced and digested in 0.5mg/ml collagenase I (Sigma-Aldrich, USA), followed by being filtered across a 70 μm cell strainer. Tissue blocks trapped in the cell strainer were seeded and cultured in DMEM/F12 supplemented with 15% FBS (Gibco, USA). Primary CAFs were identified based on morphology, FCM analysis, and immunofluorescence, which were stained positive for FAP (fibroblast activation protein) and α-SMA (α-Smooth Muscle Actin), negative for EpCAM, CD45 and CD31. Primary fibroblasts from the first to the sixth passages were used in the following experiments.

### CAR design and viral vector production

**Figure 1:**
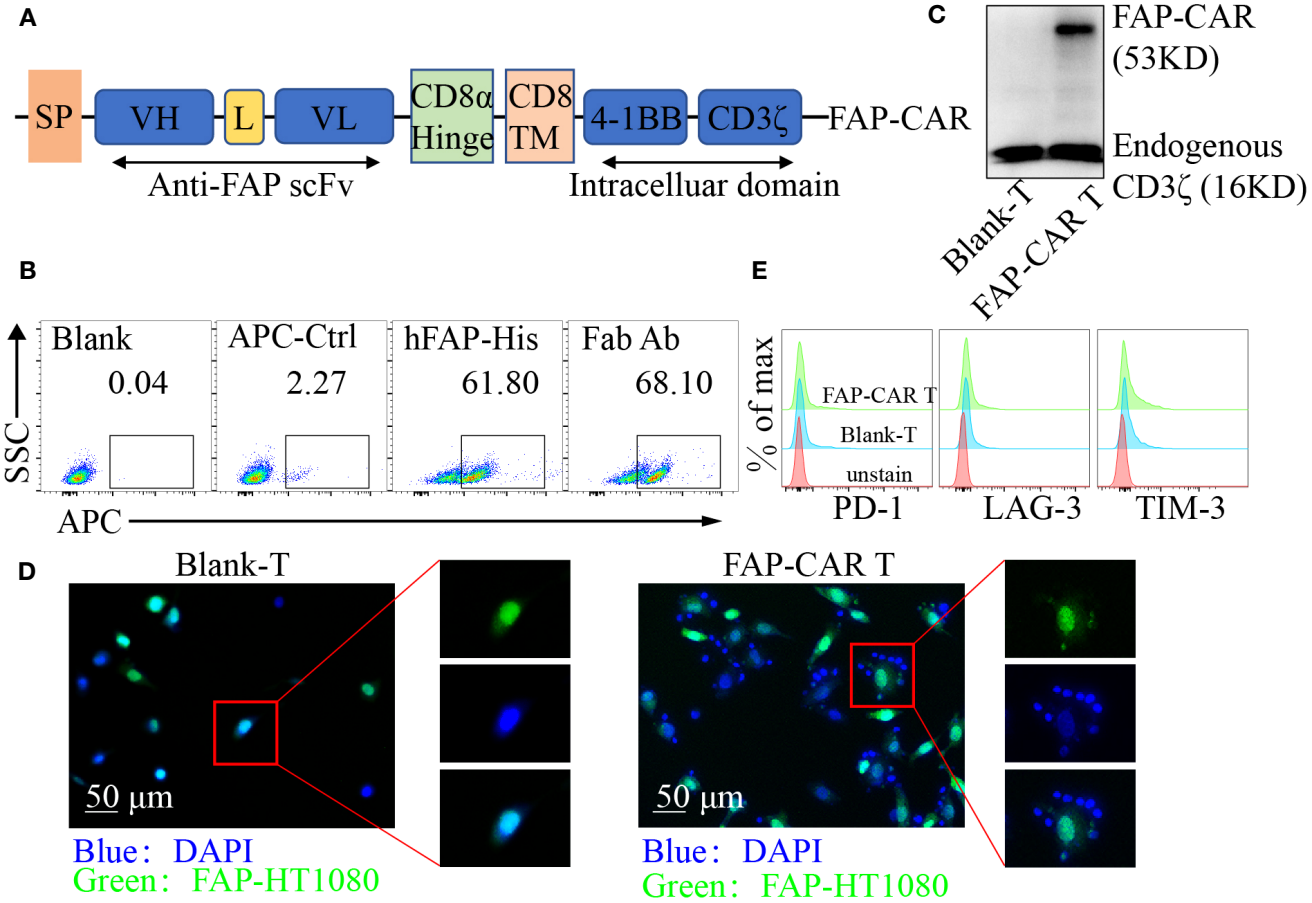
Identification of hFAP-CAR T cells *in vitro*. **(A)** Schematic representation of FAP-based CAR constructs containing CD8α signal peptide, anti-FAP scFv, the CD8α hinge and transmembrane region, and 4-1BB intracellular signaling domains, fused to the cytoplasmic region of the CD3ζ. **(B)** FCM analysis and **(C)** Western blot identifying FAP-CAR expression on T cells. **(D)** hFAP-CAR T cells recognized and linked the hFAP-HT1080 cells (EGFP^+^FAP^+^). hFAP-HT1080 cells were incubated with T cells for 90 min. Then cells were fixed and stained with DAPI. Rosette formation was detected by confocal microscope. **(E)** Exhaustion marker expression on hFAP-CAR T cells 10 days after initial activation.

The chimeric FAP CAR composed of FAP scFv and CD8α signal peptide, hinge region, transmembrane region, and cytoplasmic domain combinations of 4–1BB and CD3ζ, as shown in **Figure 1A**. The *FAP scFv* was modified and synthesized according to previous report (20). FAP-CAR lentiviruses were produced by transfecting 293T cells with the lentiviral packaging vector according to our published protocol (21). In brief, 293T cells were transfected with CAR plasmids together with the packaging plasmids using Lipofectamin 3000 Transfection Reagent (Invitrogen, USA). The culture supernatants were harvested 72h later. Virus solutions were filtered through 0.45 μm filters and concentrated by ultracentrifugation (HITACHI, Japan).

### Generation of CAR-T cells

PBMCs were cultured adherently for 12 h, and suspension cells were collected for sorting. The suspension cells were mixed with anti-CD3/CD28 Dynabeads (Gibco, USA) for 90 min, followed by separation using magnetic separator (Beaver Biomedical, Suzhou, China). Murine splenic T cells were stimulated with anti-CD3 and anti-CD28 (clone 145–2C11 and 37.51) for 24 hours. After being transduced with FAP-CAR lentivirus, cells were cultured in fresh media containing IL-2 (100 IU/ml) for 7-10 days.

### Western blot

Whole-cell lysates were prepared as previously described (22). Equal amounts of protein were resolved by 10% SDS–PAGE. Transferred proteins were probed with primary antibodies, followed with HRP-conjugated secondary antibody. The positive immune reactive signal was detected by ECL (Fude Biotech, Hangzhou, China). Antibodies specific for CD3ζ, GAPDH and β-actin were purchased from Proteintech, for FAP and α-SMA from Abcam (Cambridge, UK), for p-AKT, anti-AKT, ERK and p-ERK from Cell Signaling Technology (Danvers, MA, USA).

### Flow cytometry

All samples were analyzed using a BD FACSVerse (BD Biosciences), and data were analyzed using FlowJo 10 software. Flow cytometry staining protocol has been previously described (21). The transduction efficiency of FAP-CAR in T cells was detected by staining T cells with Alexa Fluor 647‐conjugated goat anti‐mouse IgG, F(ab′)2 fragment-specific antibody (Jackson Immunoresearch, West Grove, PA, USA), or with His-tagged human FAP (10464-H07H-B, Sino Biologicals, Beijing, China) followed by AF647-conjugated His antibody. Tumor infiltrating cells were analyzed using antibodies including APC anti-hFAP (427819, R&D Systems), FITC anti-huCD3 (UCHT1, Biolegend), APC anti-huCD3 (UCHT1, Biolegend), APC-Cy7 anti-huCD4 (RPA-T4, Biolegend), BV510 anti-huCD8 (RPA-T8, Biolegend), PE-Cy7 anti-huCD69 (FN50, BD Biosciences), PerCP-Cy5.5 anti-huCD25 (M-A251, Biolegend), APC anti-huCD107a (H4A3, Biolegend), PE-Cy7 anti-huPD1 (EH12.1, BD Biosciences), PE anti-huLAG3 (11C3C65, BD Biosciences), BV421 anti-huTIM3 (RMT3-23, BD Biosciences), PE anti-huGranzyme B (GB11, Biolegend), PE anti-huCD44 (IM7, BD Biosciences), APC anti-huCD62L (DREG-56, Biolegend), PerCP-Cy5.5 antihuCD31 (WM59, Biolegend), PE anti-hu EpCAM/CD326 (9C4, Biolegend), FITC anti-huCD45 (HI30, Biolegend), FITC anti-mEpCAM (G8.8, Biolegend), APC anti-mFAP (983802, R&D Systems), PerCP-Cy5.5 anti-mCD31 (390, BD Biosciences) and APC-Cy7 anti-mCD45 (I3/2.3, Biolegend). Intracellular staining of granzyme B was performed using fixation/permeabilization solution kit (BD Biosciences). Foxp3 and Ki-67 staining were performed using transcription factor buffer set (BD Biosciences).

### Cytotoxicity assay

T cells were co-cultured with target cells at E:T ratio of 1:1, 5:1, and 10:1 for 6-8 h. The supernatant was detected for LDH release using the CytoTox96 Non-Radioactive Cytotoxicity Assay (Promega, USA) according to the manufacturer’s instruction.

### Cytokine detection

The production of IL-2, IFN-γ, and TNF-a were measured by the Cytometric Bead Array (CBA) Human Th1/Th2 Cytokine Kit II (BD Biosciences, San Jose, USA) according to the manufacturer’s instructions. The results were analyzed using FCAP Array software version 3.0 (BD Biosciences).

### Rosette test

The hFAP-HT1080 cells (EGFP^+^) mounted onto sterile round coverslips were co-cultured with hFAP-CAR T cells (or Blank-T cells) at 4°C for 90 min. After thorough washes, the mixed cell populations were fixed and stained with DAPI (Servicebio, China). Then the round coverslips were mounted onto glass slides with antifade reagent and observed using a confocal laser scanning microscope (Olympus, Japan).

### CFSE cell proliferation assay

The hFAP-CAR T cells or Blank-T cells (5×10^5^ cells/well) were labeled with CFSE using the CFSE Cell Division Tracker Kit (Biolegend) and then cocultured with 5×10^5^ hFAP-HT1080 cells for 72 h. The intensity of CFSE fluorescence was quantitated using flow cytometry.

### *In vivo* experiments

Animal experiments were approved by the Ethics Committee of Tongji Medical College of HUST. Female NOD/ShiLtJGpt-*Prkdc* ^em26^ *Il2rg* ^em26^/Gpt (NCG) mice and BALB/c mice, C57BL/6 mice aged 4-6 weeks were purchased from GemPharmatech (Nanjing, China).

For the hFAP-HT1080 xenograft model: 1×10^6^ hFAP-HT1080 cells were injected subcutaneously into the axilla. Ten days later, the mice were randomly divided into hFAP-CAR T, Blank-T and PBS groups. 5×10^6^ human T cells or same volume of PBS were injected into the tail vein on days 0 and 7. If tumor size in one mouse reached approximately 2000 mm^3^, the experiments were terminated. Mice were injected with ^68^GA-FAPI-04 by tail vein and imaged using a micro-PET/CT scanner (Beijing Yongxin Medical Equipment Co., Ltd, China).

For the 4T1 orthotopic allograft model: BALB/c female mice were implanted orthotopically in the right 4th mammary fat pad with 1×10^5^ 4T1 cells. After 14 days, mice were injected intraperitoneally with 100 mg/kg cyclophosphamide and with 5×10^6^ murine T cells in the tail vein the next day.

For the Panc02 allograft model: C57BL/6 female mice were subcutaneously inoculated with 2×10^6^ Panc02 cells. When tumor volume reached 50-100mm^3^, mice were injected intraperitoneally with 100 mg/kg cyclophosphamide and with 5×10^6^ murine T cells in the tail vein the next day.

For the patient-derived xenograft (PDX) models: Surgically removed liver tumor tissues were cut into 5 × 5 × 5 mm^3^ pieces and quickly embedded into Matrigel prior to being transplanted subcutaneously into the flank of NCG mice to develop the PDX model (P0 mice). When the size reached 1000 mm^3^, tumor was dissected, cut in small pieces (5 × 5 × 5 mm^3^) and re-grafted into new generation of mice (P1 mice). When the tumor volume reached 250 mm^3^, P1 mice were injected with 5 × 10^6^ CAR-T cells or controls on days 0 and 7.

After being sacrificed, mice organs were harvested for histopathologic analyses. Plasma alanine aminotransferase (ALT), aspartate aminotransferase (AST), Alkaline Phosphatase (ALP) and Amylase (AMY) were analyzed using corresponding assay kits (Nanjing Jiancheng Bio-engineering Institute, Nanjing, China). Non-esterified fatty acid (NEFA) was assayed using Amplex Red Free Fatty Acid Assay Kit (Beyotime Biotechnology, Haimen, China).

### Histopathological examination

Major organs and tumors were collected and fixed in 4% paraformaldehyde for 24 hours. Paraffin sections were used for hematoxylin and eosin (H&E) staining for the preliminary safety evaluation of CAR-T cells *in vivo*. For the immunohistochemical (IHC) imaging, paraffin sections were incubated with antibodies specific for human CD3 (Proteintech, China) overnight at 4°C. Then sections were further stained with secondary antibody (HRP-labeled goat anti-rabbit IgG) at room temperature for 30 min, followed by incubation with 3,3’-diaminobenzidine (DAB, Beyotime, Hangzhou, China) for 5 min. Finally, sections were dehydrated in a gradient of alcohol and observed under the optical microscope after being sealed with neutral resin. All images were examined by a professional pathologist.

### Statistical analysis

Data are presented as the mean ± standard errors of the means. Student’s unpaired t-test was used to compare statistical differences between groups, and one-way ANOVA or two-way ANOVA was used for comparison among multiple groups. A *p* value of less than 0.05 was considered significant. All statistical analyses were performed using Prism software, version 8.0 (GraphPad, Inc., San Diego, CA, USA).

## Results

### hFAP-CAR T cells are successfully established

FAP is highly expressed on CAFs in a variety of solid tumors and is relatively poorly expressed in normal tissues (23), making FAP an attractive target in CAR-T cell immunotherapy. To establish FAP-redirected T cells, the *FAP scFv* gene was synthesized and modified according to published report (20). Surface plasmon resonance (SPR) analysis showed that the affinity of the scFv with hFAP protein was around 14.7 nM (**Supplementary Figure 1A**), comparable with 8-9 nM of parental FAP scFv (MO36) (20). Accordingly, a lentiviral vector containing the FAP-recognizing scFv, a CD8 transmembrane structural domain, a CD3ζ T cell activation structural domain, and 4-1BB costimulatory structural domains was constructed as previously described (**Figure 1A**) (21). After transduction, around 61.80% cells were detected as hFAP-His-positive and 68.10% as murine F(ab′)_2_-positive (**Figure 1B**). Western blot for CD3ζ in hFAP-CAR T group exhibited a band with the expected molecular weight of 53 kDa, along with the band of 16 kDa endogenous CD3ζ (**Figure 1C**). To investigate whether the hFAP-CAR T cells could specifically recognize and bind with hFAP^+^ cells, suspension hFAP-CAR T cells were co-cultured with adherent hFAP-HT1080 cells (EGFP^+^FAP^+^). If engaging with adherent cells, suspension T cells would also adhere to the culture surface and not be removed by thorough washes. Immunofluorescence results showed that the hFAP-HT1080 cells cocultured with hFAP-CAR T cells formed the rosette-like configurations, but with Blank-T cells not (**Figure 1D**), proving the recognition and binding capacity of hFAP-CAR T cells with hFAP^+^ cells.

Transduced T cells could expand more than 100-fold after 10 days of culture, still with high expression of Ki-67 (**Supplementary Figures 1B, C**) and no obvious alteration for the exhaustion marker PD-1, LAG-3 and TIM-3 levels (**Figure 1E**), as well as for the frequencies of CD8^+^ T, Tregs and Tcm subsets, when compared with blank-T cells (**Supplementary Figures 1D–F**).

### hFAP-CAR T cells are potent in killing hFAP^+^ targets *in vitro*

**Figure 2:**
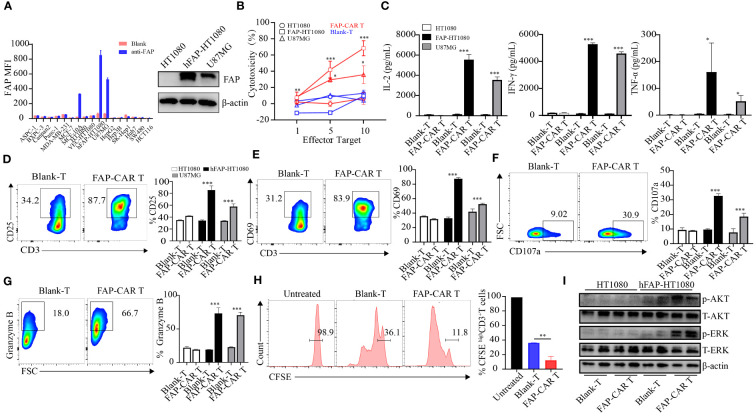
The constructed CARs mediate specific responses to target antigens. **(A)** hFAP expression level on various cell lines and on hFAP-HT1080 cells. **(B)** Cytotoxicity of hFAP-CAR T cells evaluated by LDH assay (E/T ratio = 1:1, 5:1, 10:1, 8h). **(C)** Cytokines released by FAP-CAR T cells (E/T ratio = 5:1, 24h). **(D)** CD25, **(E)** CD69, **(F)** CD107a and **(G)** Granzyme B expression on T cells after co-culture with target cells (E/T ratio = 5:1) for 24 (h) The representative FCM graphs show T cells co-cultured with FAP-HT1080 cells. **(H)** Proliferation of FAP-CAR T cells determined by the CFSE dilution assay (E/T ratio = 1:1, 72h). **(I)** Activation of Erk-Akt pathway in hFAP-CAR T cells (E/T ratio = 10:1, 6h). Results are representative of three independent experiments performed with CAR T cells derived from three different donors. The column chart shows mean ± SD, n ≥3. *P < 0.05, **P < 0.01, ***P < 0.001.

In that most of the tumor cells themselves rarely expressed FAP, hFAP overexpressed cell line FAP-HT1080 was established (16) for the subsequent test (**Figure 2A**). When CARTs were co-cultivated with targets, they elicited cytotoxicity against hFAP-HT1080 and U87MG cells in a E/T ratio-dependent manner, but not against FAP-negative HT1080 cells. And the cytotoxicity of CARTs was correlated with the FAP level, as they exhibited the highest lytic capacity against hFAP-HT1080 cells among FAP differentially expressed cell lines (**Figure 2B**). Above phenomena were confirmed by more release of cytokines (including IL-2, IFN-γ and TNF-α), upregulation of activation markers (including CD25, CD69, CD107a) and elevated level of granzyme B by CARTs when exposed to hFAP-positive target cells (**Figures 2C–G**), as well as more central memory T cells (Tcm) differentiating into effector memory T cells (Tem) (**Supplementary Figures 1G, H**).

In line with the activation of CARTs, their proliferation ability was also stronger than that of Blank-T cells (**Figure 2H**). As AKT and ERK signaling are implicated in T-cell activation and growth (24), the phosphorylation levels of AKT and ERK in T cells were detected. Results showed that more AKT and ERK were phosphorylated in hFAP-CAR T cells after co-culture with hFAP-HT1080 than with HT1080 (**Figure 2I**).

Taken together, these results demonstrated that hFAP-CAR T cells could specifically mediate cytotoxicity to targets in FAP dependent manner.

### hFAP-CAR T cells are cytolytic to human primary CAFs

**Figure 3:**
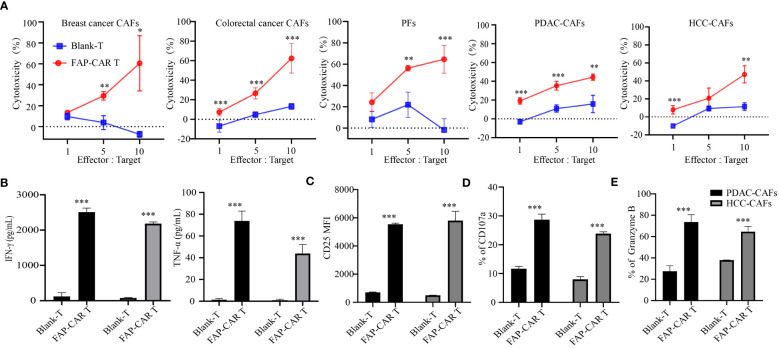
hFAP-CAR T cells are cytolytic to primary CAFs *in vitro*. **(A)** Cytotoxicity of hFAP-CAR T cells to primary cancer-associated fibroblasts (CAFs), including breast cancer CAFs, colorectal cancer CAFs, pancreatic tumor fibroblasts (PFs), pancreatic ductal adenocarcinoma CAFs (PDAC-CAFs) and hepatocellular carcinoma CAFs (HCC-CAFs). **(B)** Cytokines released by FAP-CART cells (E/T ratio = 5:1, 24h). **(C)** CD25, **(D)** CD107a and **(E)** Granzyme B expression on T cells after co-culture with PDAC-CAFs/HCC-CAFs (E/T ratio = 5:1) for 24 (h) The results are presented as the mean and SD from experiments that are performed in triplicates. *P < 0.05, **P < 0.01, ***P < 0.001.

Given that FAP is predominantly expressed in CAFs (23), human primary CAFs from several tumor types were isolated and their molecular characterization was identified as being negative for leukocyte (CD45), endothelial (CD31), epithelial (CD326), positive for hFAP, α-SMA and with an elongated spindle-like morphology (**Supplementary Figure 2A**). Results manifested that these CAFs from breast cancer, colorectal cancer, pancreatic ductal adenocarcinoma (PDAC) and hepatocellular carcinoma (HCC), as well as immortalized pancreatic tumor fibroblasts (PFs), were all substantially lysed by hFAP-CAR T cells but not by blank-T cells, in a E:T ratio dependent manner (**Figure 3A**). Accordingly, CARTs secreted more IFN-γ, TNF-α, granzyme B and expressed more CD25, CD107a than blank-T cells when exposed to PDAC-CAFs, HCC-CAFs (**Figures 3B–E**) and PFs/breast cancer CAFs/colorectal cancer CAFs (**Supplementary Figures 2B–E**).

### hFAP-CAR T cells effectively inhibit hFAP^+^ xenograft tumor growth in CDX and PDX models

**Figure 4:**
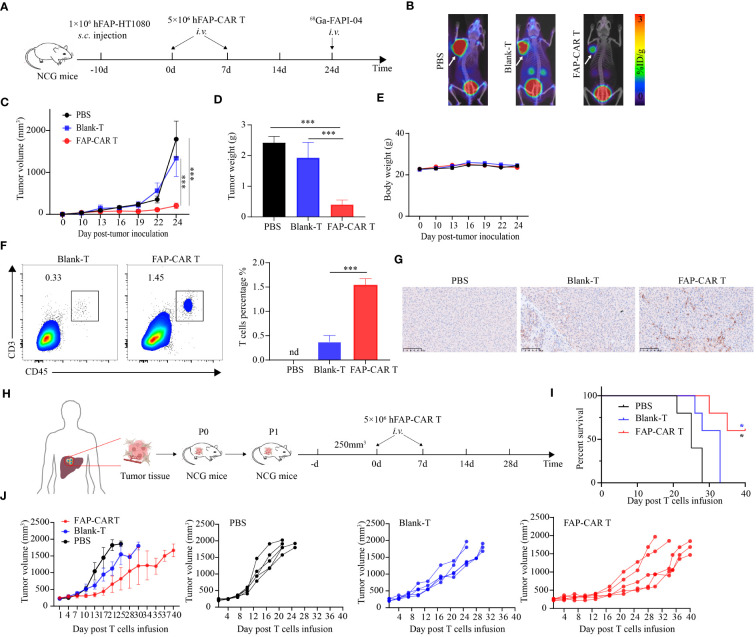
hFAP-CAR T cells effectively inhibit the progression of FAP^+^ tumors and PDX model tumors *in vivo*. **(A)** Flow chart of hFAP-HT1080 subcutaneous xenograft model. **(B)** Tumor progression was imaged using ^68^Ga-labeled FAPI-04 PET/CT, the white arrow in the picture indicates the tumor. **(C)** Tumor growth curves. **(D)** Tumor weight and **(E)** body weight. **(F)** Frequencies of tumor infiltrating hFAP-CAR T cells. Representative FCM plots (left) and statistical analysis (right) were shown. **(G)** Immunohistochemical analysis for CD3^+^ T cell infiltration in tumor tissues. **(H)** Flow chart of PDX model. P1 generation NCG mice were treated with hFAP-CAR T cells. **(I)** Kaplan- Meier curve showing the percent survival of PDX mice. Log- rank test was used for statistical analysis. **(J)** Tumor growth curves. All data are presented as mean ± SD, n=5-6. *P < 0.05, ***P < 0.001.

To determine the *in vivo* efficacy of hFAP-CAR T cells on tumor progression, hFAP-HT1080 xenografted mice were established and then treated with CARTs or controls as shown in **Figure 4A**. Micro PET/CT imaging showed that ^68^GA-FAPI-04 radiotracer accumulated evidently in tumors in PBS or Blank-T treated mice but weakly in hFAP-CAR T cells treated ones (**Figure 4B**). Consistent with the imaging results, tumor growth was inhibited in mice treated with FAP-CAR T cells (**Figures 4C, D**). There was no significant difference in the body weight between the three groups (**Figure 4E**). The frequencies of tumor infiltrating CD45^+^CD3^+^ cells were increased from 0.38% ± 0.06% in Blank-T group to 1.55% ± 0.07% in FAP-CARTs group (**Figure 4F**). And this increment was verified by immunohistochemical staining which detected more CD3^+^ cells present in CARTs treated tumors (**Figure 4G**). These data demonstrated that hFAP-CARTs can suppress hFAP-HT1080 tumor progression *in vivo*.

To better simulate the microenvironment of the originating tumor, a patient-derived xenografts (PDX) model was established using patient hepatic cancer tissue to further evaluate the anti-tumor effects of hFAP-CAR T cells (**Figure 4H**). Similar with hFAP-HT1080 xenograft model, adoptive transfer of hFAP-CAR T cells prolonged the survival time of PDX model with 60% mice still alive 40 days after treatment, while all other controls dead within 32 days (**Figure 4I**). And tumor grew less quickly in hFAP-CAR T cells group than in controls (**Figure 4J**). Because human hepatic cancer cells lack the expression of FAP (**Figure 2A**), combining with the cytolytic potential of hFAP-CAR T cells to primary CAFs from several tumor types (**Figure 3**), above data could suggest that it was through eliminating microenvironmental CAFs that FAP-CAR T cells slowed tumor growth in the PDX model. The above results demonstrated that hFAP-CAR T cells could effectively inhibit the growth of hFAP^+^ xenograft tumors in CDX and PDX models.

### mFAP-CAR T cells are also specific to murine CAFs

**Figure 5:**
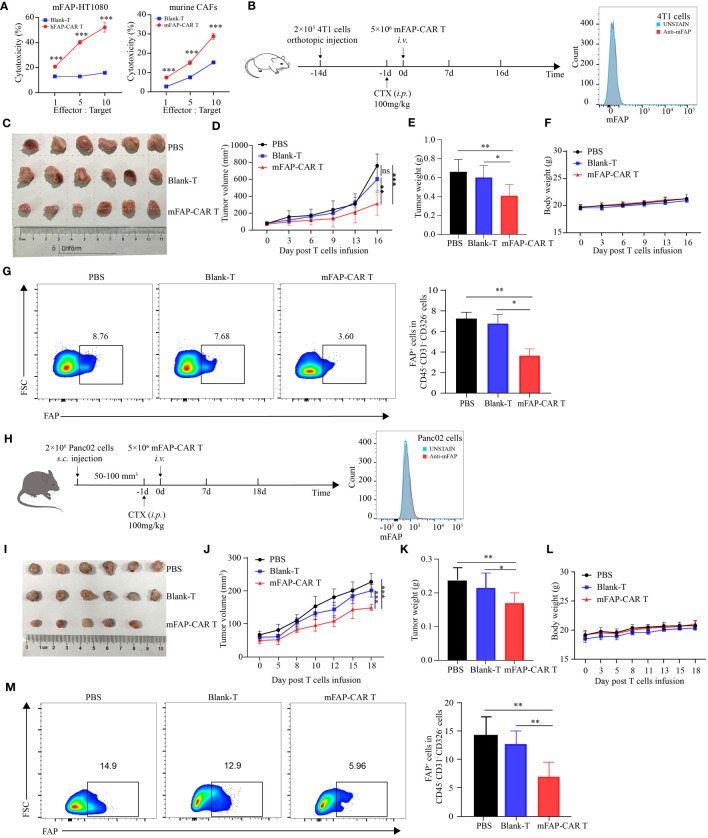
mFAP-CAR T cells are also specific to mouse CAFs. **(A)** Cytotoxicity of hFAP-CAR T cells to mFAP-HT1080 (left) and cytotoxicity of mFAP-CAR T cells to murine CAFs (right). **(B)** Flow chart of 4T1 orthotopic allograft model (left) and mFAP expression on 4T1 cells (right). **(C)** The images of allograft tumors with different treatment modalities. **(D)** The volume change of tumor in mice after murine T cells infusion. **(E)** The tumor weight was measured on day 16 post T cells infusion. **(F)** Changes in body weight of mice after T cells infusion. **(G)** Percentage of FAP^+^ tumor-associated cells. Representative FCM plots (left) and statistical analysis (right) were shown. **(H)** Flow chart of Panc02 allograft model (left) and mFAP expression on Panc02 cells (right). **(I)** The images of allograft tumors with different treatment modalities. **(J)** The volume change of tumor in mice after T cells infusion. **(K)** The tumor weight was measured on day 18 post T cells infusion. **(L)** Changes in body weight of mice after T cells infusion. **(M)** Percentage of FAP^+^ tumor-associated cells. Representative FCM plots (left) and statistical analysis (right) were shown. All data are presented as mean ± SD, n=5-6. *P < 0.05, **P < 0.01, ***P < 0.001, ns, not significant.

Above experiments verified the potential of FAP-targeted CARTs in lysing human FAP positive carcinoma and CAFs *in vivo*. In that the parental FAP-specific scFv is cross-reactive for human and murine FAP (20), it brings convenience for us to establish more murine models to evaluate the therapeutic potential of the CARTs in multiple types of tumors. To verify the cross-reactivity of FAP-CAR T cells, murine FAP-overexpressed HT1080 cells (mFAP-HT1080) and Panc02 cells (mFAP-Panc02) were established and murine CAFs were enriched from KPC mouse model. Data showed that all types of mFAP^+^ cells were sensitized to death by treatment with CARTs, but not with Blank-Ts, supporting the murine specificity of the FAP-CAR T cells (**Figure 5A**). In the following *in vivo* experiments, mFAP-negative 4T1 (**Figure 5B**) and Panc02 cells (**Figure 5H**) were allografted into BALB/c and C57BL/6 mice, respectively. It was observed that 4T1 mice slowed their tumor progression after being transferred with mFAP-CAR T cells (**Figure 5C**). The gross tumor volumes in Blank-Ts and PBS groups exhibited no difference during observation period, but were much larger than those in murine CARTs group at day 16 (**Figure 5D**), with heavier tumor weight (**Figure 5E**) and less mFAP^+^ tumor-associated cells than in CARTs group (**Figure 5G**). There was no significant difference in the body weight between the three groups (**Figure 5F**). In Panc02 allografted C57BL/6 mice, similar phenomena were observed (**Figures 5H–M**). These data confirmed that murine FAP-CAR T cells were also specific to murine (including BALB/c and C57BL/6) CAFs. Given this fact, it could not be excluded that the tumor suppression effect of CARTs was partially conferred by elimination of murine host stroma in hFAP-HT1080 xenograft model and PDX model (**Figure 4**).

### FAP-CAR T cells are biologically safe and exhibit low-level on-target off-tumor toxicity

The results in **Figures 4** and **5** confirm that our FAP-CAR T cells are cross-reactive to human and murine FAP-positive carcinoma and CAFs. This species-cross reactivity allows us to utilize mice model to somewhat predict clinical on-target off-tumor toxicity (OTOT) of the FAP-CAR T cells in the treatment of solid tumors. Due to the differences in human and mouse antigen structure, target antigen expression patterns and different tissue microenvironments, it is necessary to be cautious in predicting clinical nontumor toxicity in mice. Therefore, we can only detect as many indicators as possible in the existing model to reflect the safety and OTOT effect of CARTs.

**Figure 6:**
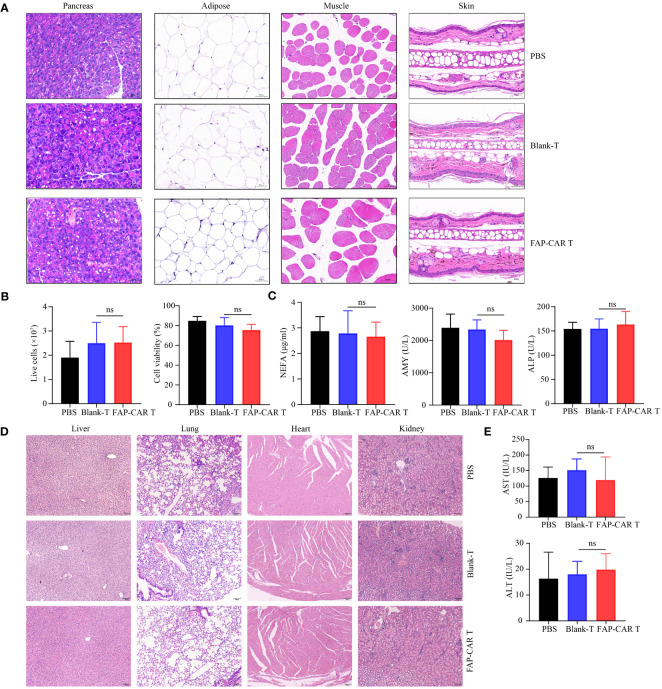
Evaluation of OTOT effect and safety of FAP-CAR T cells therapy. **(A)** H&E staining of pancreas, adipose tissue, muscle and skin tissue. **(B)** Changes in the number and activity of bone marrow cells. **(C)** Level of plasma Non-esterified fatty acid (NEFA), Alkaline Phosphatase (ALP) and Amylase (AMY) in Panc02 mice model. **(D)** H&E staining of liver, lung, heart, and kidney. **(E)** Levels of plasma alanine aminotransferase (ALT) and aspartate aminotransferase (AST) in hFAP-HT1080 xenograft model. All data are presented as mean ± SD. ns, not significant.

In that FAP is also expressed at low basal levels in healthy tissues including muscle, adipose tissue, pancreas, skin, and bone marrow (25), to evaluate the OTOT, above organs and tissues were isolated from Panc02 model mice 18 days after T cells therapy. Histological staining showed that, compared with controls, no significant histological changes were found in above tissues after mice were treated with mFAP-CAR T cells (**Figure 6A**). And there were no evident alterations in the number and activity of bone marrow cells (**Figure 6B**). The serum NEFA (derived from adipose tissue), ALP (derived from several sites including the liver, bone, intestine, and kidneys), and AMY (derived from pancreas) were at comparable levels among three groups (**Figure 6C**), with myoglobin (derived from muscle) not detectable (data not shown). These results indicated our mFAP-CAR T cells mediated low-level on-target off-tumor toxicity to model mice.

To test the biological safety of CARTs product, blood sera and major organs including liver, lung, heart and kidney in xenografted NCG mice were collected. H&E staining showed that, following T cells infusion, the mice did not develop a progressively worsening disease characterized by significant pathological changes in these major organs (**Figure 6D**). And there was no significant difference in AST and ALT levels among the three groups of mice, which were all within the normal range for NCG mice (**Figure 6E**).

Collectively, these results suggested that FAP-CAR T cells were potent in killing human and murine FAP^+^ cells and were biologically safe and exhibited low-level toxicity.

## Discussion

Generating FAP-CAR T cells that target CAFs has been an attractive therapeutic approach. In addition to eliminating their powerful and diverse protumoral effects, an advantage to targeting CAFs is that they are more genetically stable than tumor cells, so they are less likely to lose antigen expression via immunoediting. Moreover, since mesenchymal tumoral stromal cells are present in almost all human adenocarcinomas, therapies against CAFs could be used for multiple types of tumors (26). In our study, considering the restrictive expression of FAP, HT1080 cells overexpressing FAP (hFAP-HT1080) was applied to verify that the hFAP-CAR T cells could lyse hFAP-positive tumor cells specifically *in vitro* and *in vivo*. However, expression of FAP by malignant cells is limited to a few cancer types. In following *in vitro* experiments, heterogeneously originated CAFs from breast cancer, colorectal cancer, pancreatic ductal adenocarcinoma and hepatocellular carcinomas, were tested to be sensitive to cell death by hFAP-CAR T cells *in vitro*, broadening our FAP-CAR T cells use.

Although CAFs are less likely to lose antigen expression via immunoediting, it has been validated that they have plasticity. The gradual evolution of CAFs *in vivo* may lead to their differentiation into various subpopulations that promote tumor progression in both tissue-specific and tumor-specific manners (9). In this study, our FAP-CAR T cells could slow the tumor growth in multiple types of tumors in different mice models, including liver cancer PDX NCG mice models, 4T1 allograft BALB/c models and Panc02 allograft C57BL/6 models, warranting their wide application prospect in clinical practice.

In hFAP-HT1080-derived and liver cancer patient-derived xenograft models, it was concluded that hFAP-CAR T cells inhibited tumor progress by eliminating human FAP-positive tumor cells or stroma cells, in line with other reports showing that FAP-redirected T cells can inhibit tumor growth by depleting FAP^+^ stromal cells or tumor cells (7, 27–29). However, different from those reports using scFv against only human FAP, in this paper, human murine cross-reactive FAP scFv was applied. In the CDX model, human xenografts need the support from murine stromal cells for at least, the structural maintenance of tissues. And in patient-derived xenografts (PDX) model, human stromal cells originally present in tumors dissected from patients are gradually replaced by host stromal cells as the xenograft grows (30). Turtoi Andrei et al. even reported that in liver metastasis PDX model, human stroma was entirely replaced at the second generation although PDX maintained original tumor architecture and the metabolic profiles of both stromal and cancer cells remained stable for at least four generations (31). Hence, in our human xenograft mice models, we could not exclude the possibility that above effect was also conferred by elimination of murine host stroma. The cross reactivity of FAP-CAR T cells brings us convenience to somewhat investigate the contributions of CAFs ablation to tumor progression in allografted mice models, and need not to use conventional xenograft mouse models in which human cancer cells with human CAFs are co-injected (5). In FAP-negative 4T1 and Panc02 allografted models, infused FAP-CAR T cells can only recognize and therefore lyse tumor stromal fibroblasts to slow the tumor progression, proving deletion of CAFs a valuable tumor therapeutic approach.

The FAP-CAR T cells has been reported to inhibit tumor progression in an immune-response dependent manner in several tumor models (26). For instance, Steven M Albelda et al. reported that the mouse FAP-CAR T cells had no antitumor effects on AE17.ova tumors in the immunodeficient NSG mice. They suggested that FAP^+^ cells depletion in murine tumors might enhance antitumor immunity by initially activating endogenous T cells, followed by increasing intratumor T cell infiltration at a later timepoint (27). Recently, it has also been reported that FAP-CAR T cells could deplete stromal cells and matrix-dense network surrounding tumor nests, thus disrupted immune exclusion. The destruction of the matrix enhanced the motility of CAR T cells and made it easier for them to cross the tumor boundary and enter the tumor nest (32). In our human xenograft models, FAP-CAR T cells did not lose activity in the NCG tumor microenvironment, suggesting that through elimination of FAP-positive stroma cells could FAP-CAR T cells limit the tumor growth. However, FAP-CAR T cells were supposed to have stronger antitumor effects in immune-competent mice than in immunodeficient mice, because their efficacy could be observed as early as 16 days after CARTs administration in wild-type mice, while in NCG mice, they need to spend more time. It’s worthy of our further investigation on how FAP^+^ cells orchestrate immune cell function using more desmoplastic, non-immunogenic mouse tumor models and human xenografts. Anyway, the synergistic effect with endogenous T-cell antitumor responses would promise a better efficacy of FAP-CAR T cells in clinic setting.

The cross reactivity of FAP-CAR T cells also allows us to somewhat predict the clinical OTOT of the FAP-CAR T cells in mice. FDA guidelines for preclinical animal testing of cellular therapies require the use of a relevant animal model capable of eliciting a biological response that would reasonably predict the expected human response (33). However, in many cases, animal models used for pharmacological testing of therapeutic human CARTs cannot adhere to this standard due to variability in cross-species reactivity to nonhuman target antigens (34). Because of lacking expression of hFAP on normal mouse tissue, it’s difficult for CARTs specific for hFAP to predict their off-tumor targeting in animal studies. For our FAP-CAR T cells, they could recognize both human and murine FAP, permitting the assessment of OTOT in mouse studies. Results indicated that our CARTs mediated low-level toxicity against non-malignant tissues expressing FAP in model mice, predicting that the FAP-CAR T cells would be safe and lead to low clinical OTOT in the treatment of solid tumors. But it is still necessary to be cautious in predicting clinical nontumor toxicity in mice.

In our study, FAP-CAR T cells could not eliminate tumors totally and only inhibit tumor growth in a limited period. This may result from inefficient infiltration, exhaustion or poor persistence of CARTs (35). However, trogocytosis is another consideration. It was found that only 6 hours after co-culture with FAP-CAR T cells, hFAP-HT1080 cells lost parts of their surface FAP expression (data not shown), in line with recent reports that tumor cells decreased their target density after contact with CARTs (36, 37). One explanation is that the FAP-CAR T cells may extract surface FAP from the conjugated tumor cells through immunological synapse (38). The active transfer of FAP to the CARTs would abate T cell activity by promoting fratricide T cell killing and T cell exhaustion, and lead to escape of antigen-low tumors. Hence, measures should be taken in future studies to augment tumor responses to FAP-CAR T cells by cooperative killing and combinatorial targeting, for instance, blockade of PD-1 (39–41), combination therapy of dual-target CAR-T both targeting CAFs and tumor cells (29, 42).

In addition to its high expression in tumor tissues, FAP expression is up-regulated in a variety of fibrotic diseases, including hepatic fibrosis (43), pulmonary fibrosis (44, 45), and myocardial fibrosis (46). It has been reported that adoptive transfusions of FAP-CAR T cells can significantly reduce cardiac fibrosis and restore its impaired function in mice (46). In our study, it was also found that the expression of FAP in IPF was significantly up-regulated. Therefore, we are exploring the possibility of FAP-CAR T cells in the treatment of pulmonary fibrosis and its mechanism of action, and corresponding progress has been made, which will be presented in subsequent articles. At present, the OTOT effect of CAR T cells has been paid much attention. Due to the differences in human and mouse antigen structure, target antigen expression patterns and different tissue microenvironments, it is necessary to be cautious in predicting clinical nontumor toxicity in mice. The safety and OTOT may be only be better evaluated in higher-level animal models or clinical studies, which is under our consideration. Therefore, we can only detect as many indicators as possible in the existing model.

Taken together, the human/murine cross-reactive FAP-CAR T cells were powerfully potent in killing human and murine FAP-positive tumor cells and CAFs. They were biologically safe and exhibit low-level OTOT, warranting further clinical investigation into our FAP-CAR T cells.

## Data availability statement

The original contributions presented in the study are included in the article/**Supplementary Material**. Further inquiries can be directed to the corresponding authors.

## Ethics statement

The studies involving humans were approved by the ethical standards of the Medical Ethics Committee of the Tongji Medical College, Huazhong University of Science and Technology. The studies were conducted in accordance with the local legislation and institutional requirements. The participants provided their written informed consent to participate in this study. The animal study was approved by Animal Care Ethics and were approved by Tongji Medical College of Huazhong University of Science and Technology. The study was conducted in accordance with the local legislation and institutional requirements.

## Author contributions

WN: Methodology, Software, Writing – original draft, Formal analysis, Project administration. BW: Methodology, Software, Writing – original draft. YZ: Formal analysis, Methodology, Writing – original draft. CW: Methodology, Writing – original draft. JC: Software, Writing – original draft. JL: Software, Writing – original draft. YH: Funding acquisition, Resources, Supervision, Validation, Writing – review & editing. PL: Investigation, Supervision, Validation, Writing – original draft, Writing – review & editing.

## Supplementary material

Supplementary Figure 1
**(A)** The affinity of scFv to hFAP protein was measured by Biacore surface plasmon resonance. **(B)** Expansion of hFAP-CAR T cells. **(C)** Ki-67 expression in hFAP-CAR T cells 10 days after initial activation. The percentages of **(D)** CD4^+^/CD8^+^ subsets and **(E)** Tregs in hFAP-CAR T cells. Memory phenotype of FAP-CAR T cells **(F)** before and **(G, H)** after exposure to targets (E/T ratio = 5:1, 20h). Naïve: CD44^-^CD62L^+^; central memory: CD44^+^CD62L^+^, CM; effector memory: CD44^+^CD62L^-^, EM. The column chart shows mean ± SD, n ≥ 3.

Supplementary Figure 2 Identification of CAFs. Primary CAFs were isolated from tumor masses of breast cancer, colorectal cancer, pancreatic ductal adenocarcinoma, and hepatocellular carcinoma. **(A)** CAFs were identified as CD45^-^CD326^-^CD31^-^FAP^+^(right), immunofluorescence detection for α-SMA expression on CAFs (left). **(B)** Cytokines released by hFAP-CAR T cells (E/T ratio = 5:1, 24h). **(C)** CD25, **(D)** CD107a and **(E)** Granzyme B expression on T cells after co-culture with pancreatic tumor fibroblasts (PFs), Colorectal cancer CAFs and Breast cancer CAFs (E/T ratio = 5:1) for 24 h. All data are presented as mean ± SD. ****P* < 0.001.

Supplementary Figure 3 mFAP-CAR T cells are cytolytic to mFAP^+^ Tumor cells *in vitro*. **(A)** Expression of mFAP in mFAP-Panc02 cells. **(B)** Cytotoxicity of mFAP-CAR T cells to Panc02 cells (left) and mFAP-Panc02 cells (right). **(C)** The expression of CD25 on T cells after co-culture with target cells (E/T ratio = 10:1) for 24 h.
