# Supplementary figures and images for: Efficacy and safety evaluation of cross-reactive Fibroblast activation protein scFv-based CAR-T cells
Source: Front Immunol. 2024 Jul 17;15:1433679. doi: 10.3389/fimmu.2024.1433679 (PMC11288799; doi:10.3389/fimmu.2024.1433679)

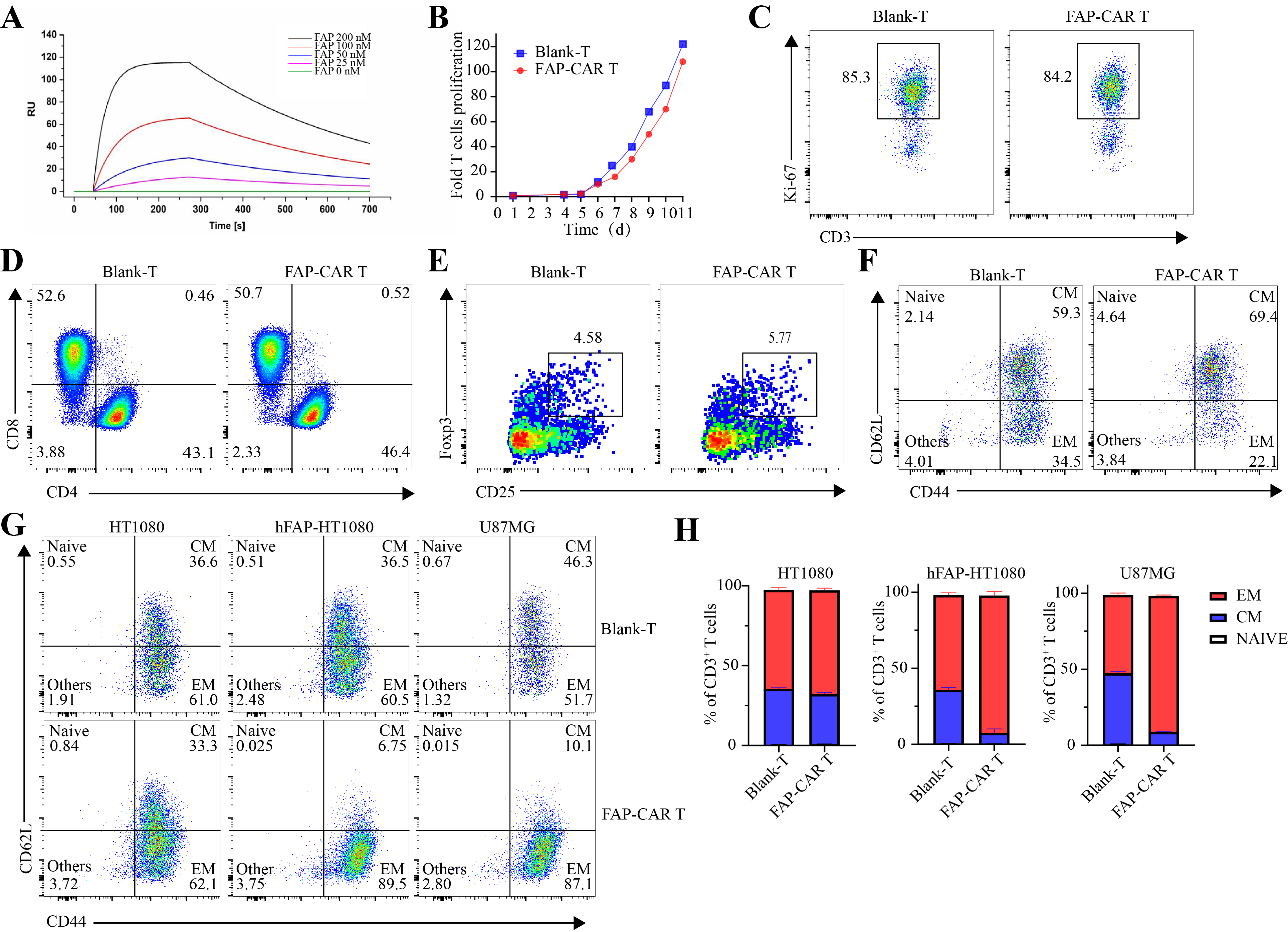

Supplement: Supplementary Figure 1 — (A) The affinity of scFv to hFAP protein was measured by Biacore surface plasmon resonance. (B) Expansion of hFAP-CAR T cells. (C) Ki-67 expression in hFAP-CAR T cells 10 days after initial activation. The percentages of (D) CD4+/CD8+ subsets and (E) Tregs in hFAP-CAR T cells. Memory phenotype of FAP-CAR T cells (F) before and (G, H) after exposure to targets (E/T ratio = 5:1, 20h). Naïve: CD44-CD62L+; central memory: CD44+CD62L+, CM; effector memory: CD44+CD62L-, EM. The column chart shows mean ± SD, n ≥ 3. [file Image_1.jpeg]

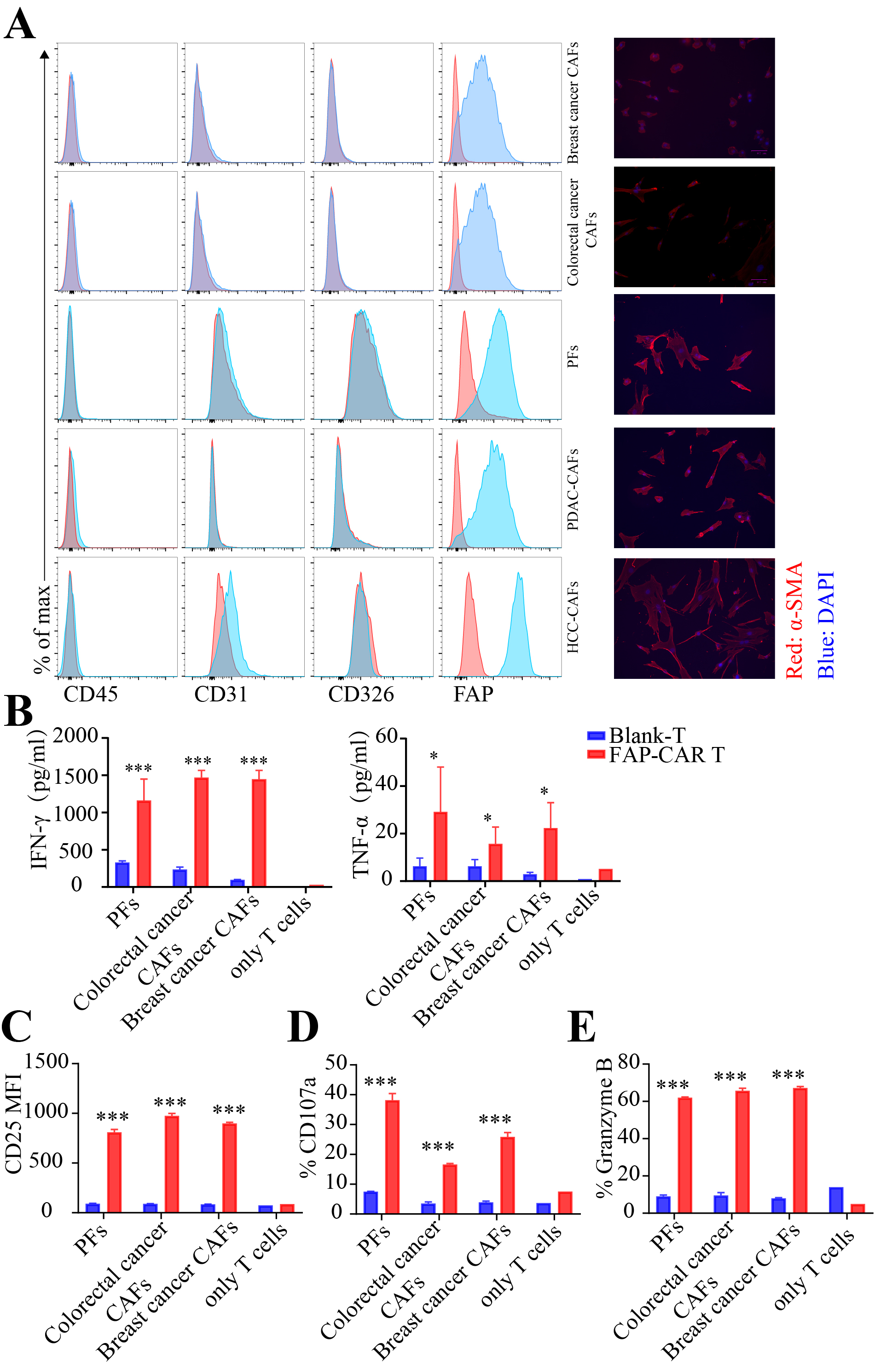

Supplement: Supplementary Figure 2 — Identification of CAFs. Primary CAFs were isolated from tumor masses of breast cancer, colorectal cancer, pancreatic ductal adenocarcinoma, and hepatocellular carcinoma. (A) CAFs were identified as CD45-CD326-CD31-FAP+(right), immunofluorescence detection for α-SMA expression on CAFs (left). (B) Cytokines released by hFAP-CAR T cells (E/T ratio = 5:1, 24h). (C) CD25, (D) CD107a and (E) Granzyme B expression on T cells after co-culture with pancreatic tumor fibroblasts (PFs), Colorectal cancer CAFs and Breast cancer CAFs (E/T ratio = 5:1) for 24 h. All data are presented as mean ± SD. ***P < 0.001. [file Image_2.jpeg]

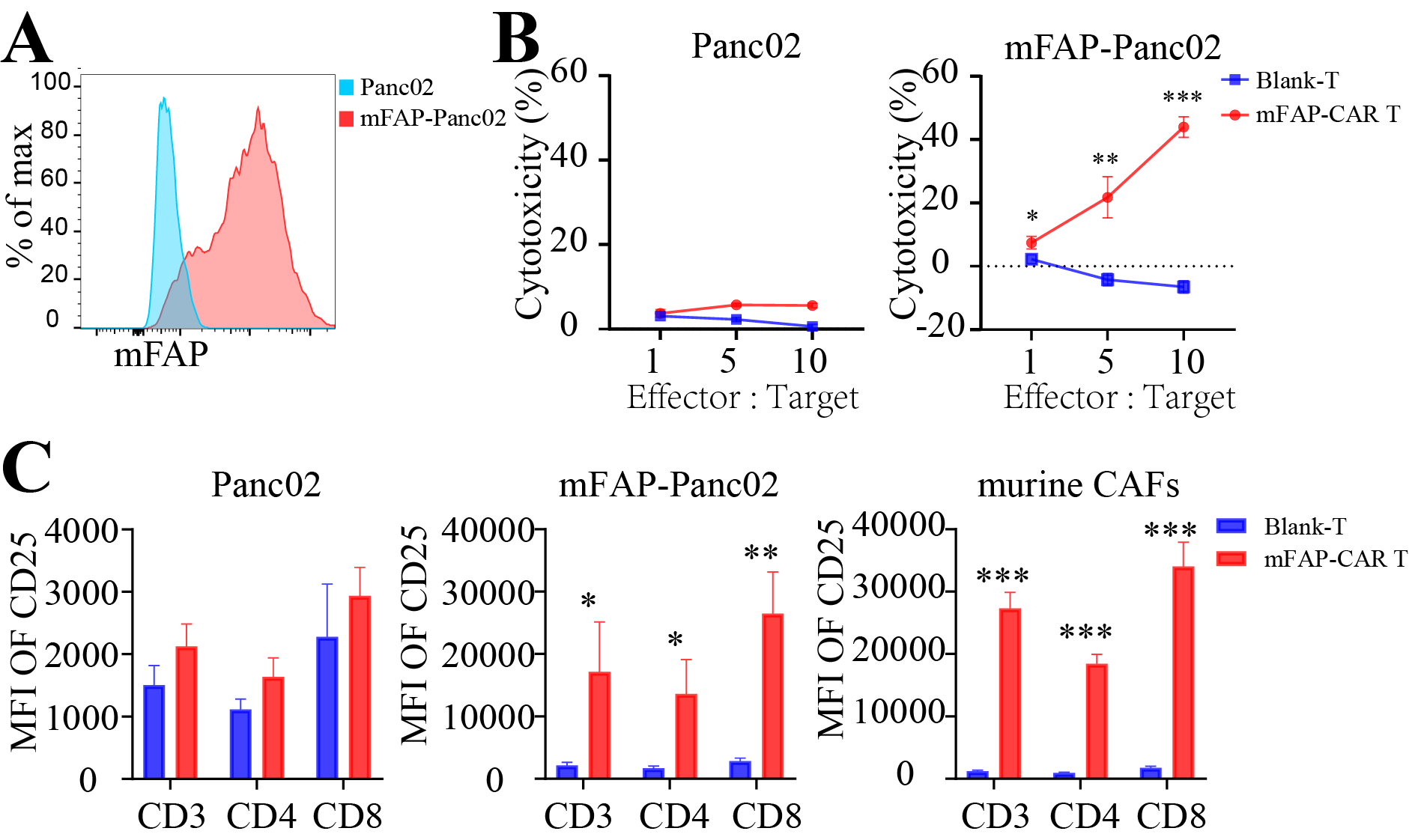

Supplement: Supplementary Figure 3 — mFAP-CAR T cells are cytolytic to mFAP+ Tumor cells in vitro. (A) Expression of mFAP in mFAP-Panc02 cells. (B) Cytotoxicity of mFAP-CAR T cells to Panc02 cells (left) and mFAP-Panc02 cells (right). (C) The expression of CD25 on T cells after co-culture with target cells (E/T ratio = 10:1) for 24 h. [file Image_3.jpeg]
